# Training for Foodborne Outbreak Investigations by Using Structured Learning Experience

**DOI:** 10.3201/eid2601.190755

**Published:** 2020-01

**Authors:** Florian Burckhardt, Esther Kissling

**Affiliations:** European Programme for Intervention Epidemiology Training Alumni Network, Heidelberg, Germany (F. Burckhardt);; Epiconcept, Paris, France (E. Kissling)

**Keywords:** epidemiology, food safety, foodborne disease outbreaks, disease outbreaks, outbreak investigations, symptoms, *Salmonella* species, training support, structured learning experience, disease detective cards

## Abstract

We created a free and interactive training activity based on playing cards (disease detective cards) that introduces foodborne outbreak investigations to public health professionals and students. Competencies taught cover selected descriptive and analytic epidemiologic topics, such as case definition, epidemic curve, 2-by-2 tables, relative risks, attack rates, stratification, and confounding.

Training programs, such as the Epidemic Intelligence Service of the Centers for Disease Control and Prevention (Atlanta, GA, USA) (*1*) and the European Programme for Intervention Epidemiology Training of the European Centre for Disease Prevention and Control (Stockholm, Sweden) (*2*), teach future epidemiologists outbreak investigation skills. Their case studies for foodborne outbreaks are often based on historic investigations whose real-world complexity can be overwhelming for beginners.

An introductory study (*3*) used 14 exposures to explain the concept of risk factors and their association with disease. We designed a scripted foodborne outbreak investigation as a structured learning experience with illustrated playing cards that depict exposures (food eaten) and outcome (symptoms, time to first symptoms). With the cards and a flipchart as props, facilitators teach core skills and competencies of outbreak investigations in an interactive group exercise.

We modeled a hypothetical *Salmonella* species outbreak investigation with a cohort design in Excel 2010 (Microsoft, https://www.microsoft.com). We took symptoms and incubation periods from our own unpublished outbreak investigations and literature research (*4*,*5*). We reverse-engineered relative risks for univariable and stratified analysis with their 2-by-2 tables into a line list with the desired properties and transcribed onto playing cards in which each card represents 1 person attending the exposure event.

Dynamic illustrations of gastroenteric symptoms and food items were hand drawn with pen and ink on Bristol board, scanned, and colored by using the GNU Image Manipulation Program (https://www.gimp.org). The illustrations depict characters from our earlier work, the disease detectives (*6*). We used OpenOffice software (https://www.openoffice.org) for layout of the cards. The teaching script is a restructured and simplified adaptation of outbreak case studies used during European Programme for Intervention Epidemiology Training introductory courses (*2*,*3*). The cards and other resources are available (https://www.disease-detectives.org) under a Creative Commons open-culture license in multiple formats for home printing or as templates for high-quality printing services.

The exercise is for 16–26 students who play roles of participants in the outbreak event and investigate their own outbreak. Each participant draws 1 card that illustrates their fictional health status, food eaten, symptoms if ill, and time of onset (Figure). Facilitators subsequently collect the information from the cards and summarize them on a board during the 1–2-hour teaching activity as the outbreak investigation unfolds. The facilitator should have professional experience in investigating foodborne outbreaks, accompanying statistical methods, and adult learning. Their role is to tap into experiences of the participants and weave them into the narrative of the fictional outbreak investigation. Depending on the learner audience and their skills, the facilitator can use a lecture style or an activity-centered style with small group discussions and collaborative summaries (*7*). The exercise is designed to work without electricity and only a board or flipchart. It covers the skills of developing a case definition, collecting data for preparing an epidemic curve, and calculating relative risks as a measure of strength of association between exposure and outcome.

**Figure:**
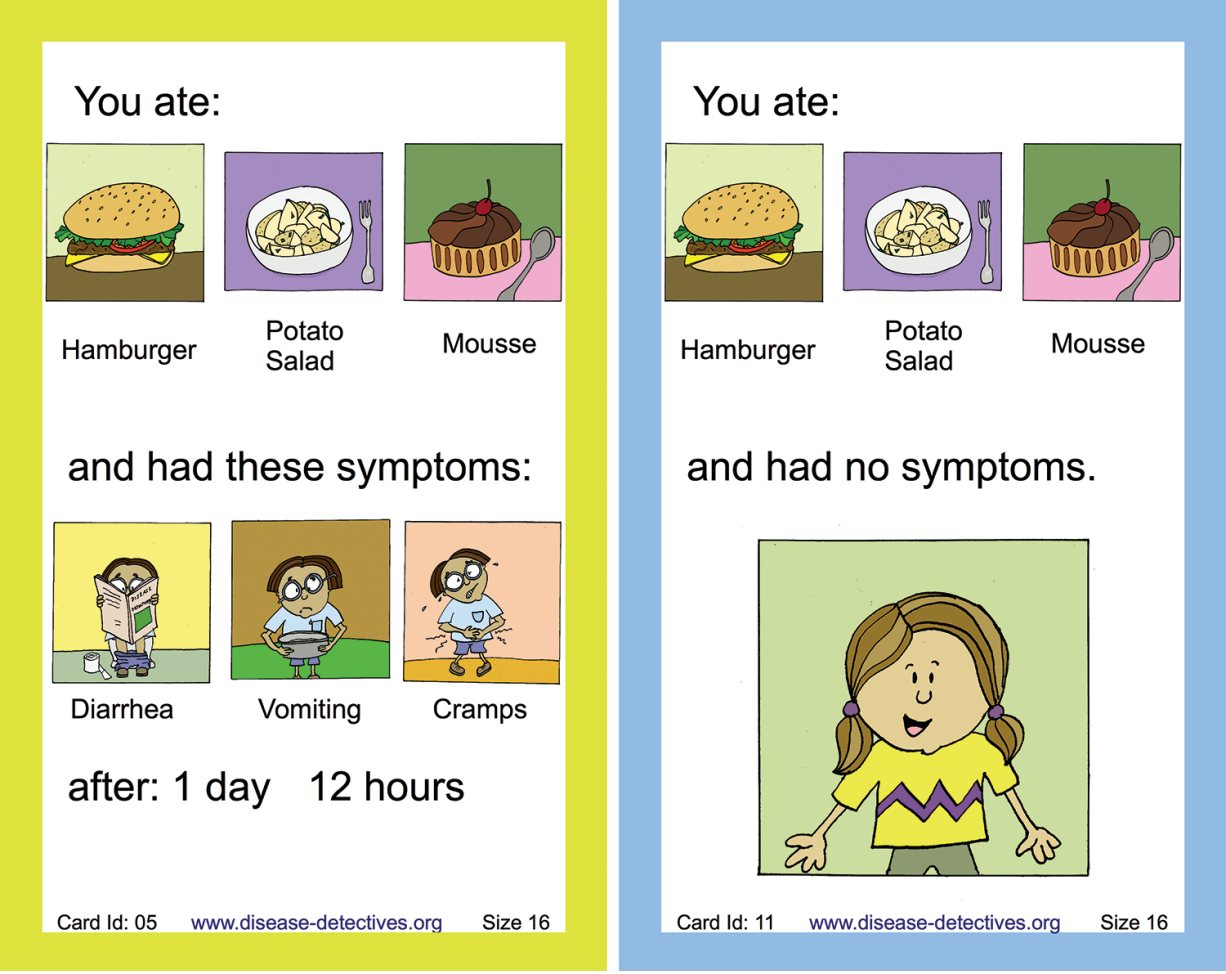
Playing cards used to describe symptoms, their timing, and food eaten as exposure for training for foodborne outbreak investigations by using structured learning experience.

In addition, exposure-specific attack rates and cases explained by exposure, stratification, and confounding can be introduced at the facilitators’ discretion. The teaching script suggests the sequence of introducing epidemiologic concepts and collection of relevant data from the cards of individual participants. The exercise is limited to the epidemiologic descriptive and analytic part of an outbreak investigation and does not cover environmental investigation, such as trace back or cover laboratory methods, results, or interpretation. The card model can be extended to train other outbreak patterns (e.g., norovirus) with more vomiting as a symptom and a 2-peak epidemic curve caused by secondary human-to-human exposure.

Since 2014, we have used the teaching activity in 4 district health authority trainings and 1 training of hospital hygiene doctors in Rhineland-Palatinate, Germany. The German Federal Office of Consumer Protection and Food Safety in Berlin used the activity in 2 training sessions on outbreak investigations during 2018, and the Robert-Koch Institute in Berlin used the cards in their outbreak investigation module of an annual training for public health professionals in infectious disease surveillance and during Kids Day (*8*).

Source material can be modified to adapt the exercise to different cultural contexts. During training workshops in Sudan and Tunisia, under the German Biosecurity Programme of the Federal Foreign Office during 2016 and 2018 (*9*), hamburger and potato salad were substituted with shawarma and salade niçoise as more culturally appropriate food for northern Africa. The interactive card training tool had been positively reviewed in the training evaluation comments, and participants remained engaged throughout the session. We are planning a formal evaluation of the training tool with the field epidemiology training programs for Germany and Europe.

The exercise is a tool for building outbreak response capacities and teaches the topic in an engaging way. It can be used on its own or embedded as an ice breaker into a field epidemiology curriculum for health professionals or for school classes looking for health-related project work. Supported languages are German, English, Russian, and French. Further translations and adaptations are encouraged and will be referenced on our web page (https://www.disease-detectives.org).
